# Health Inequalities, Intersectionality, and Justice

**DOI:** 10.1111/1468-0009.70133

**Published:** 2026-10-10

**Authors:** ERIKA BLACKSHER

**Affiliations:** ^1^ University of Kansas Medical Center; ^2^ University of Washington

**Keywords:** Health inequalities, health inequities, intersectionality, population health, social determinants, health equity, justice, ethics

## Abstract

**Policy Points**

- Population health science that uses large data sets to study health inequalities increasingly draws on intersectionality, the idea that overlapping forms of disadvantage shape health inequalities. This approach is essential to the pursuit of health equity because it provides a more complete and sound evidence base for population health policy.
- As this approach has gained traction, it has drifted from intersectionality's founding ideas about social power and justice.
- This Perspective develops a framework of justice for the field that consists of a multidimensional account of unjust power relations and an egalitarian standard to guide population health research and interventions.

**Context:**

Research that examines multiple forms of marginalization simultaneously, referred to herein as quantitative intersectional analysis, is an important trend in population health science. This approach can identify otherwise hidden health inequalities and a fuller array of social drivers, creating a more complete evidence base for intervention. The practice of quantitative intersectional analysis, however, has strayed from intersectionality's founding ideas about interconnecting power relations and social justice. Some researchers have called for the rebuilding of those connections.

**Methods:**

This Perspective interprets and adapt key features of Nancy Fraser's critical theory with the aim of developing a framework of justice and illustrating its value as an ethical resource for quantitative intersectional analysis and population health policy.

**Findings:**

The proposed framework is well suited to quantitative intersectional analysis in three respects. First, it's critical vantage point foregrounds precisely the kinds of power relations and social structures that are well‐documented drivers of health inequalities, including maldistribution in economic relations, misrecognition in cultural relations, and misrepresentation in political relations. Second, the framework is adaptable and can be extended, as recommended here, to include care relations. Third, the framework articulates a positive standard of justice—participatory parity—that can be interpreted to provide both moral justification for the view that some health inequalities constitute health inequities and ethical guidance for population health policy.

**Conclusions:**

The proposed framework of justice supports deep engagement with ideas about social power and justice in four ways. It expands the social categories and systems of inequality typically subject to analysis, enhances conceptual clarity and theorization about the nature of the harms under study, identifies health inequalities that merit investigation, and provides an ethical standard for population health policy that protects against the use of stigmatization as a policy tool and supports a broad and inclusive concept of health equity.

Population health science that uses large data sets to study health inequalities increasingly draws on intersectionality, the idea that overlapping and interconnecting forms of disadvantage shape the patterning of health inequalities.^1^, ^2^ Health inequalities are studied by multiple categories simultaneously (e.g., race *and* gender) rather than independently (e.g., race *or* gender) on the premise that together they condition access to social resources (e.g., education, income, housing) and exposure to social relations (e.g., racism, sexism) that become embodied and manifest in health inequalities.^2^, ^3^, ^4^ Studying health inequalities in this way “complicates everything,”^5^ but the potential return on investment of this approach is high. Quantitative intersectional analyses can identify more health inequalities than conventional single‐category studies and provide greater insight into underlying causes, creating a more complete and analytically sound evidence base for population health policy.^3^, ^5^ Some researchers consider intersectionality essential to the pursuit of health equity for these reasons.^1^, ^5^, ^6^, ^7^, ^8^

Yet as quantitative intersectional analysis has gained traction, some scholars are concerned that it has lost touch^3^, ^9^ with intersectionality's origins in Black feminist ideas about interlocking power relations and justice.^10^, ^11^, ^12^ This disconnect is seen, for example, in studies that rely on individual‐level measures (e.g., race, gender) rather than systems of inequality (e.g., racism, sexism).^13^, ^14^ Others have observed a “reductionistic”^15^ ^(p96)^ tendency to control for some characteristics in order to isolate the influence of others, a practice at odds with intersectionality's aim to explain how the interplay of multiple power relations impacts health exposures and outcomes.^16^ Researchers also disagree over which intersectional inequalities merit study.^1^, ^3^, ^17^, ^18^

These issues involve not only scientific and methodological issues but also moral ones. They entail value judgements about which forms of social power should be made visible, what constitutes an unjust form of power, and whose health burdens are worthy of investigation and intervention. These judgements need to be informed by considerations of justice.

In this article, I propose a framework of justice for quantitative intersectional analysis, drawn from the critical theory of Nancy Fraser.^19^ Although Fraser has not addressed her theory's relevance to population health or questions of health justice, others have.^20^, ^21^, ^22^ Here, I interpret the theory through the lens of population health science to develop a practical framework consisting of an intersectional account of unjust power relations and a positive standard of justice with which to evaluate when social conditions are unjust and policy solutions are ethically acceptable. I then illustrate some of the ways in which the framework can be used to deepen the field's engagement with ideas about social power and justice and sketch several policy implications. I propose the framework in the spirit of a working document, one that can be revised and refined through further theorization, empirical investigation, and interdisciplinary collaboration and debate among population health scientists, policymakers, bioethicists, and philosophers.

## Ethical Resources for Population Health Science and Policy

A resurgence of empirical interest in the social origins of disease at the turn of the 21st century spawned a groundswell of interest in questions about why health and health inequalities might be matters of justice.^23^ Theories of justice have been adapted to address such questions,^24^, ^25^, ^26^, ^27^ and definitions of health inequities have been developed to identify their morally salient features in an effort to distinguish health inequalities from health differences that are unfair, or health inequities.^28^, ^29^, ^30^, ^31^ Although such ethical resources may be informative to quantitative intersectional analysis, they also have limitations.

As Bowleg explained, widely used definitions of health inequities fail to address the intersectional nature of lived reality.^1^ The *Healthy People 2020* definition^28^ and other prominent efforts to clarify the meaning of health inequities^29^ typically identify a list of characteristics (i.e., race, ethnicity, religion, socioeconomic status [SES], gender, age, mental health, disability, sexual orientation, gender identity, geographic location) that represent forms of social disadvantage associated with health inequities. As helpful as these definitions are, they do not explain that these characteristics interconnect and interpenetrate, mutually constituting one another to form complex health inequities. Yet, this reality is key to intersectionality's promise for advancing health equity.

Definitions of health inequities also often look to distributive justice as a primary justification for the injustice of health inequalities,^29^, ^30^ which although important, is insufficient. Theories of distributive justice, which aim to secure a fair share of important goods for all, can neglect relational injustice (e.g., racism, sexism^19^) and basic respect for all^32^, ^33^; theories of relational justice, which aim to secure equal respect for all, can commit the error in reverse, neglecting distributive injustice (e.g., structural impoverishment, economic precarity).^19^ Theories of egalitarian justice more generally, in their aim to define a positive conception of justice with universal application, can abstract from real‐world social struggles, making them difficult to operationalize as a practical resource for population health science and policy. Rarely do such theories address the complexities of intersectionality head on. They also tend to ignore the profound implications of political boundaries and decision rules that determine whose voices and interests are represented in politics and policy, concerns that are central to theories of deliberative democracy.^34^ Fraser argued that any viable theory of justice meant to serve contemporary society must account for all of these moral imperatives.^34^ Fraser's multidimensional formulation of injustice, among other features, makes the theory seem purpose‐built for the purposes and practices of quantitative intersectional analysis.

## Fraser's Critical Framework of Justice

Key features of Fraser's conception of justice explain its suitability for quantitative intersectional analysis. First, Fraser takes a critical stance in theorizing about justice, foregrounding real‐world social inequalities that require evaluation and transformation.^19^, ^34^ Whereas ideal theory brackets real‐world conditions and posits a set of resources and opportunities that all people need for a good life, critical theory develops an incisive empirical account of the power dynamics and inequalities that unfairly limit people's life chances and lived realities in contemporary society.^35^, ^36^ Thus, the foci of Fraser's critical theory of justice are precisely the power relations and social structures found in many population health theories, conceptual models, and definitions of social determinants that aim to identify upstream causes and to situate health inequalities within broader sociological and historical contexts.^37^, ^38^, ^39^, ^40^

Second, the theory integrates an explicit moral analysis of what justice is with an empirical analysis of the power relations that undermine it. That moral analysis turns on participatory parity, the idea that all persons should have genuine opportunities to be and act as peers in public life.^19^ Participatory parity is the standard for establishing the requirements of justice and evaluating claims of injustice. Grounded in the equal moral worth of all persons, participatory parity requires ending relations of social subordination and establishing the institutional prerequisites (e.g., constitutions, laws, public policies, tax codes, social practices) that make participatory parity accessible to all. Moreover, participatory parity expresses a commitment not only to documenting and explaining social inequalities but to remaking the social structures and systems that unfairly limit people's life chances. Thus, the theory's ultimate egalitarian aim is to build an evidence base for social action, a motivation shared by those who argue that quantitative intersectional analysis can yield more actionable research for population health policy.^3^, ^5^

Third, Fraser's empirical analysis pinpoints three power relations that threaten participatory parity: maldistribution in economic relations, misrecognition in cultural relations, and misrepresentation in political relations. Because each form of power relation has some autonomy vis‐á‐vis the others in shaping social inequalities in modern capitalism, none is reducible to or derivative of the other. Each is a primary injustice with independent *moral* standing. Fraser's formulation of how these power relations work in the world is, however, explicitly intersectional. They are presumed to interconnect and interpenetrate in varied ways across social and political contexts that change over time. Thus, Fraser's working theory of real‐world inequality aligns with intersectional theorists and researchers who hold that there can be no predetermined ranking of power relations, no reigning “ism” (e.g., structural racism, structural sexism, capitalism) that explains all the others all the time.^5^, ^18^ The dynamics of their interplay are empirical matters for science to determine.

Fourth, all three power relations are well‐documented drivers of health inequalities, corresponding to economic, cultural, and political determinants of health, as I explain next.

### Three Health‐Relevant Dimensions of Injustice

*Maldistribution* in economic relations refers to processes of SES deprivation and exploitation rooted in capitalist economies that determine forms of property, ownership, labor, production, and consumption and create a class structure on the basis of characteristics such as income, wealth, education, and occupation.^19^ Maldistribution is manufactured by ideologies, institutions, and policies that create relations of domination on the basis of wealth, control over property and labor conditions, and opportunity hoarding based on education and credentialing that determines access to occupational positions and income from employment.^41^ These social processes fabricate conditions such as economic precarity and structural impoverishment that produce classes (e.g., precariat, underclass) subject to economic harms such as poverty wages, unemployment, under‐ and uninsurance, food insecurity, eviction, and homelessness. The health effects of maldistribution are well‐established.^42^, ^43^ No matter how it is measured (e.g., income, education, occupation, wealth, area deprivation), SES is a pervasive and persistent predictor of variations in health that, like racism,^44^ acts as a fundamental cause of health inequalities.^45^ Theories of social class that incorporate or integrate Marxist, Weberian, and Bourdieusian mechanisms and educational constructs are increasingly being developed to examine and explain this association.^46^, ^47^, ^48^

*Misrecognition* in cultural relations refers to processes of cultural interpretation and communication that treat some individuals as unworthy of equal respect and create status groups on the basis of characteristics such as race, gender, and sexuality. Misrecognition is generated by institutionalized hierarchies of cultural value that judge some as inferior, treating them in demeaning and discriminatory ways and depriving them of equal respect and shared humanity.^19^ Misrecognition may amplify differences, casting some to the outskirts of humanity, or deny differences that function as sources of respect, forcing assimilation and dismissing distinctive traditions, cultures, and histories. Paradigmatic examples include racism, sexism, and heterosexism, but misrecognition also encompass harms such as ageism, ableism, sizeism, and classism, or any “ism” based on misjudgments about the inferiority of people with certain characteristics. Whatever form it takes, misrecognition marks some individuals as different, deficient, or deviant, subjecting them to harms such as marginalization, oppression, and violence, legally sanctioned or otherwise. The health effects of some forms of misrecognition, such as racism, sexism, and heterosexism, are well‐documented or growing in evidence.^49^, ^50^, ^51^ The stigmatization to which misrecognized groups are subject is a fundamental driver of health inequities.^52^ Theories and models for measuring the health effects of various forms of misrecognition (e.g., structural racism, structural sexism, structural stigma, whiteness) continue to be developed.^14^, ^50^, ^53^, ^54^, ^55^, ^56^, ^57^ whereas others, such as classism, have yet to be modelled for such purposes.^14^

*Misrepresentation* in political relations refers to governance structures, decision rules, and political institutions and practices that discount or disappear the voices and votes of some by creating citizens and noncitizens on the basis of characteristics such as birthplace and geographic and political boundaries (e.g., state, nation, immigration status, rural or urban environment).^34^ Political institutions and practices make the rules of democracy and determine who has a voice and a vote and how much, if at all, they count—how much they influence the setting of policy agendas, the selection of candidates, and the making laws and public policies. Misrepresentation deprives people of voice, belonging, and equal representation through institutions and practices, such as voter suppression, partisan gerrymandering, and “winner take all” electoral rules. The political determinants of health have long been studied,^58^, ^59^ continue to be theorized and elaborated,^60^, ^61^ and are increasingly tested empirically. Studies have shown, for example, that strong electoral democracies (e.g., those holding free and fair elections and ensuring checks and balances in power) and left‐leaning politics are associated with longer life expectancy and lower infant mortality.^62^, ^63^, ^64^ A recent study showed an association between weak electoral democracy and rising midlife mortality at the US state level.^65^

### The Standard of Participatory Parity

The discussion thus far has established why injustice as Fraser defines it (i.e., maldistribution, misrecognition, misrepresentation) matters to health. What has not yet been established is why health matters to justice as Fraser defines it (i.e., participatory parity). The case can be made in several ways. One might view some threshold level of health as a prerequisite to participatory parity. According to this view, health and health‐protective resources enable people to become and act as full and equal members of society. Individuals cannot be so chronically debilitated by illness and bereft of health‐relevant resources that they cannot participate as peers with others in important life activities such as learning, working, loving, exercising their liberties, and participating in community and civic life. A second view holds that health is an essential component of participatory parity. According to this view, health itself should be included among the bundle of important goods that justice must secure for all, an argument that finds additional footing in the modern view that individuals should be supported in achieving health.^66^ Both views interpret health as special in relation to justice,^67^ whether as a precondition to or component of it.

A third way to interpret the matter is that health inequalities signal something unfair about society.^68^ It is not health itself that matters according to this view but rather what the social patterning of health says about social arrangements. This philosophical view is compatible with the idea of embodiment, widespread in population health, that the social patterning of health and disease reflects an unfair distribution of property, prestige, and power within and across populations.^4^ One need not choose among these perspectives. Taken together, they strengthen the case that health and its lack are matters of justice.

## Applications of the Framework

Commentators have called on researchers who conduct quantitative intersectional analyses to draw on interdisciplinary scholarship in order to deepen engagement with ideas about social power and justice.^3^, ^9^, ^13^, ^14^, ^69^ The proposed framework, summarized in Table 1, is one approach. I suggest three ways in which the framework may support that aim.

**Table 1:** A Critical Justice Framework

**Participatory Parity** All persons have a genuine opportunity to be and act as peers in public life. Relations of subordination are ended and institutional prerequisites are established that make participatory parity accessible to all. Policy solutions support participatory parity for all.				
**Injustice**	**What**	**How**	**Who**	**Social Determinants**
Maldistribution	Economic resources and educational opportunities	Economic relations that deprive, exploit, and dominate to create a class structure and hoard resources and opportunity	Classes, e.g., capital class, professional class, working class, precariat class, underclass	Socioeconomic determinants (e.g., education, income, occupation, impoverishment, no or inadequate insurance, homelessness)
Misrecognition	Respect and recognition	Intersubjective relations that oppress and discriminate to deny equal respect and shared humanity	“Others” deemed different, deficient, deviant	Cultural determinants (e.g., racism, sexism, heterosexism, ableism, sizeism, ageism, classism, credentialism, nativism)
Misrepresentation	Voice, votes, influence, and representation	Political relations that discount and disappear voices, views, and votes from formal and informal political and policy processes	Citizens, noncitizens, immigrants, the stateless	Political determinants (e.g., state residence, geography, extreme gerrymandering, voter suppression, authoritarianism, political alienation)
Maltreatment	Attachment, care, social bonds	Care relations that neglect, abuse, isolate, coerce, and manipulate	Children, dependents, the vulnerable, isolated, and lonely	Affective determinants (e.g., nurture, care, affection, love, friendship, solidarity)

### Expansion of Social Categories and Systems

Much work is underway to develop models to measure identity‐based systems of oppression (e.g., structural racism, structural sexism).^14^, ^50^, ^53^, ^54^, ^55^, ^56^, ^57^ The proposed framework does not supplant this important work but instead advances a broad conceptual scheme that expands the types of injustice that are typically subject to intersectional analysis. For example, the framework's definition of misrecognition encompasses not only racism and sexism, long the foci of intersectional studies,^17^ but also other patterns of cultural meaning and communication based on ugly stereotypes about the supposed inferiority of those with certain characteristics (e.g., ableism, ageism, sizeism, heightism, classism).

More foundationally, the proposed framework brings two additional primary injustices into the main frame of intersectional health research, namely, those rooted in the economy (maldistribution) and in politics (misrepresentation). A recent study showed that the majority of intersectional analyses included sex/gender (76.7%) and race/ethnicity (71.4%), followed by SES (33.2%) and sexual orientation (20.7%),^69^ results that support the observation that class “remains underutilized as an analytic category to explain complex social inequalities.”^11^ ^(p13)^ This neglect is notable given that SES has long been a potent predictor of health.^70^, ^71^, ^72^ Education‐based inequalities in life expectancy have been widening dramatically since the 1980s.^73^ High and rising US midlife mortality (dying between 25 and 64 years old) across all racial groups is concentrated among those who lack high school and/or college degrees.^74^, ^75^

Similarly, the framework's inclusion of misrepresentation is important in the base case, given the impact of national and local politics^76^ and policy contexts on population health. However, it is especially salient given the rise of political polarization and democratic backsliding, a glaring example of which is the US Supreme Court's 2026 decision to gut the last remaining provision of the 1965 Voting Rights Act. Given that this provision was designed to protect racial minority representation in jurisdictions with documented histories of racial discrimination, the court's decision in effect silences Black Americans in electoral politics in some jurisdictions.^77^ The health effects of extreme political polarization and democratic backsliding can be seen in state‐level variations in US life expectancy, infant mortality, and midlife mortality that are attributable to divergent state‐level policy contexts that reflect the ways in which states have moved further to the political right or left and remade policies that shape residents’ health prospects (e.g., those regarding welfare, labor, guns, health care, abortion access, the environment, immigration, civil rights).^78^, ^79^, ^80^

Because political institutions and practices determine whose identities and interests are represented in policy debates about the distribution of resources (maldistribution) and who gets equal respect (misrecognition), the importance of the misrepresentation is hard to overstate. Public policies are key levers of population health because they shape social, cultural, and economic conditions.^81^ Features of democracy (e.g., checks and balances, free and fair elections), in turn, create the terms by which those public policies are debated and decided.

An open question is whether the framework is complete. Maldistribution, misrecognition, and misrepresentation encompass a wide swath of social determinants of health, but some may be missing. Take, for example, early life adversity, which has significant effects on children's developmental health, adult health, and social and economic prospects.^82^, ^83^, ^84^ None of the three power relations sufficiently captures the nature of early life adversity, abuse, and neglect because these harms occur within the family unit, where children are completely dependent on adult caregivers to provide a safe, secure, and protective environment. Feminist philosophers have long argued that theories of justice, including Fraser's,^85^ need to incorporate the family unit and care relations because they are social structures and practices that have profound effects on life chances. Adversity and maltreatment in childhood are particularly grave injustices as children cannot be held responsible for the harms that befall them. Children can exercise no control over the environments into which they are born; they can only adapt to them. Adding this fourth dimension of injustice to the framework could help remedy the status of family relations as a neglected social determinant of health^86^ and support more routine integration of a life‐course perspective^87^ into the study of health inequalities. For all of these reasons, affective relations should be added to the framework as an integral dimension of injustice. This element of the framework should also be conceptualized broadly enough to account for other forms of social bonds and care relations (e.g., social cohesion, social support), given their significant impact on health.^88^, ^89^

### Enhancement of Conceptual Clarity and Theorizing

The framework's abstraction from particulars and positing of four fundamental types of injustice might also enhance conceptual clarity about the nature of the harms in question. Consider, for example, class‐related injuries associated with capitalism and classism. Subordination rooted in economic structures that commodify and rationalize all aspects of life, prioritize profit, and discount the future—features of neoliberal capitalism that manufacture austerity, economic precarity, and impoverishment^90^—is maldistribution. In contrast, classism is a form of misrecognition that is rooted in cultural beliefs that people who lack advanced formal education are inferior and perpetuated by a meritocratic ideology that attributes responsibility for success and failure to the individual.^91^ Classism, then, is a form of misrecognition based on misjudgments about the inferiority of people with certain characteristics. In contrast, economic precarity and structural impoverishment are forms of maldistribution rooted in economic arrangements. These injustices likely travel together and, for some, enmesh with others (e.g., racism, sexism, ableism). However, they must first be distinguished to conceptualize and investigate their entwined effects, which is the core aim of intersectionality analysis.

The framework may also support theorizing about relationships within and across these categories of harm. Consider, for example, the emergence in the 1980s of a “rural health penalty,” which refers to the worse health outcomes of rural residents compared to their urban peers.^92^, ^93^, ^94^ Rural health inequalities are a global phenomenon, and among 11 high‐income countries, they are starkest in the United States.^95^ As these inequalities are often attributed to SES factors, such as poverty and access to health care resources (maldistribution), this framework invites conceptualization and empirical investigation of how maldistribution may, for example, enmesh with intergenerational poverty and child maltreatment, the stigmatization of rural places and people (misrecognition), and processes that shape rural politics and policy contexts (misrepresentation).

To be clear, the framework does not supply the theories and models needed for researchers operationalize it. Intersectional investigations will need to develop a theory of the case, that is, how these dimensions of injustice interconnect to drive a particular health inequity for a given population in a given place and time. The framework functions instead as a broad normative framework that brings four fundamental dimensions of injustice into clear analytic focus. In doing so, it illuminates a wider range of harms than is typically considered in these studies and encourages their investigation as potentially intersecting drivers of health inequalities. The framework also makes explicit the moral grounds for judging such health inequalities to be unjust. Intersectional health researchers often claim justice as a motive for their work yet rarely define what they mean by it. The proposed framework does that work, which makes it useful in another way, namely, as a resource for thinking about which health inequalities merit study.

### Identification of Health Inequalities That Merit Study

Researchers disagree about whether all intersectional positions are of equal interest or whether the focus should be on only those characterized multiple forms of disadvantage.^1^, ^3^, ^17^, ^18^ At issue are scientific questions about the potential value of examining large population samples comprising individuals in a wide range of circumstances (advantaged, disadvantaged, combinations thereof) as well as ethical questions about which intersections should be prioritized for study on the grounds of justice. This framework speaks to the latter question and issues a broad response. Health inequalities that implicate any of the four categories of injustice are morally suspect and possibly unjust, and they merit study. Should empirical investigations show that health inequalities involve multiple power relations, as intersectionality presumes until proven otherwise,^96^ those complex health inequities fit squarely within a broad agenda of quantitative intersectional research.

A paradigmatic example of a complex health inequity is the very high burden of infant mortality on African American women with low levels of education. These women are multiply marginalized: subject to racism and sexism and the often lifelong socioeconomic hardship associated with low educational attainment, all of which appear to be implicated in the observed high infant mortality rates.^97^ However, very poor outcomes can also occur in multiply marginalized groups characterized by a mix of social disadvantage and advantage. For example, college‐educated African American women have higher rates of infant mortality than White women with a high school degree, which researchers attribute to race‐ and gender‐based discrimination, socioeconomic marginalization, and early life adversity.^97^ Also illustrative are the longevity prospects of White women who lack high school diplomas, whose life expectancy declined by 3.1 years, bringing it below that of Black women of the same age and education level.^98^ Underlying drivers likely include socioeconomic factors, such as education, employment, income, and access to health care; gender‐specific factors, such as declining rates of marriage and work‐family conflict; and early life adversity.^99^, ^100^, ^101^ These latter two cases are paradoxical^102^ in that they involve an apparent advantage (e.g., college education, White identity) that fails to deliver the expected health benefit, and they speak to one of the reasons why intersectionality is essential to the study of health inequalities. Were these measures of population health studied only by race or only by class, these very poor outcomes and their injustice would remain undocumented.

As these examples suggest, the framework's wide‐angle lens on the nature of injustice casts a wide net and is likely to identify many health inequalities as health inequities, including in White communities.^103^ There has been some exchange among researchers about whether poor White communities are now worse off than African Americans in terms mortality rates,^97^, ^104^ but that debate is somewhat misplaced. It is true that theories of egalitarian justice often place some priority on aiding the worst off, but they have different ways of interpreting what that means.^105^ What it does not mean is that only one group should be identified as the worst off at any point in time or for all time. As Amartya Sen has noted about the value of mortality data for detecting unjust social arrangements, they can “move fast enough to provide guidance on a rapidly changing economic and social situation.”^106^ ^(p23)^

The more viable and less controversial way to think about the meaning of “worst off” is in terms of a threshold that all should attain or not fall below.^107^ This threshold, sometimes referred to as a basic minimum or sufficiency,^24^, ^108^ prioritizes the worst off but counts anyone below sufficiency as being badly off. Although it may not be clear precisely what sufficiency is in every case for every health indicator, there will be many cases that are clearly insufficient. One place to start is to look at how the best off are faring in terms of the relevant health indicator (e.g., infant mortality rates, life expectancy), which provides a sense of what is socially, politically, and technologically possible.^24^ Fraser has not addressed this matter explicitly, but this approach is compatible with her theory's concern for the multiply marginalized.

The idea of a basic minimum or sufficiency is particularly apt when thinking about intersectional inequalities because of how they are formed. As products of mutually constituting systems of inequality, they are said to create composites of social disadvantage that are unique and thus impossible to compare.^5^, ^18^, ^96^, ^109^ A sufficiency standard does not require the comparison of all those who are badly off in order to identify the very worst off. It is necessary only to identify a threshold below which no one should have to live and die. A sufficiency standard can, then, help society move past an unanswerable question^109^ and toward more pragmatic questions about why so many people are so badly off and what can be done to improve their lives.

## Policy Considerations

The use of intersectionality in quantitative population health science was borne in part out of a desire to make research more actionable for policymakers and community leaders.^3^, ^5^ Use of this framework may support actionability in three ways. First, research that includes measures of misrepresentation may identify levers of change that are amenable to political and civic action by an ecosystem of actors. When research identifies that health‐protective policies are needed or existing policy needs reform, philanthropies and advocacy leaders can help shape the narrative, community leaders can spread the word, people can lobby their representatives and cast votes, and policymakers can act in the people's interest. When research identifies features of a weakening democracy, other actions can be taken. Some are upstream structural reforms to political institutions and practices.^110^ Others are downstream strategies of civic advocacy, agitation, and resistance.

Second, research that incorporates forms of injustice across several of the framework's dimensions may yield considerable insight into the underlying drivers of a particular health inequality. To be effective, policies need to fit the problem, and multifaceted problems require multifaceted solutions. For example, consider rising midlife mortality, which, as noted, has been documented in those who lack college degrees in all racial groups, affecting different groups (race, gender, geography) in different ways (i.e., drug and alcohol abuse, suicide, and cardiometabolic diseases). A suite of upstream and downstream policies will be needed to address this complex population health challenge, which has tap roots in all of the framework's dimensions: the economy, culture, care, and politics.

Participatory parity can act as a standard for assessing the ethical acceptability of policies, yet a third way in which the framework supports actionability. Participatory parity requires that the pursuit of a fair distribution not come at the cost of misrecognition.^20^, ^111^ To illustrate, consider one facet of the challenge of midlife mortality. Policies will need to improve people's economic prospects in ways that do not shame or stigmatize them, which is likely in egalitarian projects.^31^, ^32^ One policy that does this is the elimination of requirements for a four‐year college degree for jobs that do not require one, as several states have done.^112^ Removing credentialist and classist barriers to employment for those who are able to do the work demonstrates respect for people from varied backgrounds, including those from poor families who had no pathway to college. However, policies will need to do more to remove barriers to work rooted in other forms of misrecognition (e.g., racism, sexism, ableism). Policies must support participatory parity for all below the threshold. They should not lift the prospects of some individuals while lowering them for others.

In this requirement, the standard of participatory parity supplies moral justification for an approach to population health policy called “proportionate universalism,” which recommends universal policies at the structural level to promote overall health and targeted policies that meet the specific needs of subpopulations.^113^ It also acts as a check against false narratives that some people must fare worse in order for others to fare better. Thus, the standard of participatory parity could protect against a “zero‐sum” mindset that has long undermined society's prospects of prospering together.^114^

## Conclusions

As intersectionality has made inroads into quantitative population health science, some worry that its use has lost touch with intersectionality's founding ideas about social power and justice. The proposed justice framework described herein offers one approach to rebuilding those connections. It describes four types of unjust social power rooted in economic, cultural, political, and care relations, all of which are documented drivers of health inequalities. It also articulates a robust egalitarian standard of justice, namely, participatory parity, to assess dynamic social conditions and societal interventions.

I propose this framework as a living document, one that is open to revision and refinement in response to further theorization and empirical investigation, as well as interdisciplinary collaboration and debate among researchers, bioethicists, philosophers, and policymakers. Whatever its weaknesses, this framework does much that commentators say needs doing. It brings critical theory^13^ to the task of advancing a broad “conceptual scheme that illuminates the political, economic, and social processes”^5^ that drive health inequalities; it foregrounds power relations, helping to counteract methodological individualism;^6^, ^13^, ^14^, ^69^ and it expands the categories typically subject to intersectional analysis. It also does something essential that can get overlooked: It makes explicit the moral grounds for judging some health inequalities to be unjust, and therefore, it can support population health scientists and policy makers in their thinking about which inequalities to study and remedy.

The framework's wide‐angle lens on the nature of injustice may identify health inequities that afflict many people in many places, including in White communities. Thus, the framework could help build a “health equity tent” that is bigger and more inclusive than is sometimes perceived.^115^ That big tent may help people from many backgrounds understand that the language and action of health equity and justice is *for* them, not against them.

## Funding/Support

The author received no financial support for the research or authorship of this article.

## Conflict of Interest Disclosures

The author has no conflict of financial or nonfinancial interests to declare.
